# Low connectivity compromises the conservation of reef fishes by marine protected areas in the tropical South Atlantic

**DOI:** 10.1038/s41598-019-45042-0

**Published:** 2019-06-14

**Authors:** Clarissa Akemi Kajiya Endo, Douglas Francisco Marcolino Gherardi, Luciano Ponzi Pezzi, Leonardo Nascimento Lima

**Affiliations:** 1 National Institute for Space Research (INPE), Remote Sensing Division, São José dos Campos, 12227-010 Brazil; 2 Euro-Mediterranean Center on Climate Change (CMCC), Ocean Modeling and Data Assimilation Division, Bologna, 40127 Italy

**Keywords:** Conservation biology, Ecological modelling, Conservation biology

## Abstract

The total spatial coverage of Marine Protected Areas (MPAs) within the Brazilian Economic Exclusive Zone (EEZ) has recently achieved the quantitative requirement of the Aichii Biodiversity Target 11. However, the distribution of MPAs in the Brazilian EEZ is still unbalanced regarding the proportion of protected ecosystems, protection goals and management types. Moreover, the demographic connectivity between these MPAs and their effectiveness regarding the maintenance of biodiversity are still not comprehensively understood. An individual-based modeling scheme coupled with a regional hydrodynamic model of the ocean is used to determine the demographic connectivity of reef fishes based on the widespread genus *Sparisoma* found in the oceanic islands and on the Brazilian continental shelf between 10° N and 23° S. Model results indicate that MPAs are highly isolated due to extremely low demographic connectivity. Consequently, low connectivity and the long distances separating MPAs contribute to their isolation. Therefore, the current MPA design falls short of its goal of maintaining the demographic connectivity of *Sparisoma* populations living within these areas. In an extreme scenario in which the MPAs rely solely on protected populations for recruits, it is unlikely that they will be able to effectively contribute to the resilience of these populations or other reef fish species sharing the same dispersal abilities. Results also show that recruitment occurs elsewhere along the continental shelf indicating that the protection of areas larger than the current MPAs would enhance the network, maintain connectivity and contribute to the conservation of reef fishes.

## Introduction

Reef fish fauna of the South Atlantic oceanic islands and coastal shelf are known to have a high rate of endemism (c.25%^1^) that has contributed to shape the biogeographical patterns observed today. The Amazon river outflow and the vast distances separating the east and central Atlantic act as barriers separating pairs of sister species. There are also differences in faunal structure of reef fishes between the continental shelf and oceanic islands in the tropical and subtropical South Atlantic due to insular isolation^2^. Evidence of this isolation can be found in results from DNA sequencing that support the hypothesis of allopatric speciation for the diversification of the *Sparisoma* genus in the Atlantic^3^.

The conservation of separate reef fish populations should maximize the demographic connectivity, defined as the exchange of offspring between populations by larval dispersal, including large-scale movements^4^. However, it is not easy to establish the actual dispersal abilities of reef fishes, since these may vary due to several biological and physical traits. Such variability has been found in the plankton larval duration (PLD, the maximum length of time that larvae can stay in the plankton) that varies depending on the species and on the environmental conditions^3^. For some commonly found species in the Atlantic, the PLD may vary from 47 to 93 days. For example, the PLD for *S. viride* has been reported to be on average between 48.3^5^ and 57^3^ days, but it may last up to 80 days^3^; for *S. radians* the average is 60 days, ranging from 50 to 93 days^3^, and for *S. aurofrenatum* the average PLD is 48.6 days^5^. Besides the PLD, the demographic connectivity is also sensitive to important physical processes over short time scales, such as the water temperature, the three-dimensional velocity field and the seasonal and interannual ocean variability that interacts with the biological traits to determine the connection pathways of eggs and larvae from different populations.

The global trend of declining reef habitats and the impact of overfishing on small populations of reef fishes have added more pressure on marine protected areas (MPAs) as they play an important role in the resilience of these populations. Until recently, the percentage of spatial coverage of MPAs in the Brazilian Economic Exclusive Zone (EEZ) was at 2%^6,7^, whilst the recommendation by the Convention on Biological Diversity targeted at 10% by 2020 (Aichi Biodiversity Target 11)^8^. It became clear that the representativeness of MPAs should be improved in the short term regarding their number, size and geographical location. Besides, the distribution of MPAs in Brazil is still unbalanced in terms of the protection goals, use, and management categories^7,9,10^. There is a lack of no-take MPAs in reef-fish hotspots for total richness, endemic/threatened species and functional groups^11^, such that only 0.8% of the total, or 51,181.2 km^2^, are no-take areas. As an obvious consequence, these no take areas are highly interspaced and susceptible to low connectivity conditions^11^. Recently, the Brazilian government increased the total MPA coverage in the EEZ from 1.5% to 25%^12^. The new coverage expanded the radius of protection around the São Pedro and São Paulo Archipelago and the Vitoria-Trindade seamount chain to 200 nautical miles creating larger MPAs^12,13^.

Despite the difficulty to accommodate a diverse set of conservation goals, MPAs need to address some basic issues to become minimally effective, which can be broadly grouped into three main categories: biodiversity pattern, demographic connectivity and resilience against ecological regime shifts due to climate change (e.g. global warming)^14^. Our present knowledge about reef ecosystems in the tropical Atlantic and their main functional groups, endemicity and threatened species has been constantly improving^15–17^ and demographic connectivity studies using biophysical models have contributed to the creation of more robust MPA networks^18–20^. Nonetheless, there is a scarcity of dedicated research to determine demographic connectivity based on the hydrodynamics and the individual behaviour of eggs and larvae in the tropical South Atlantic^21–23^.

The surface flows in the north-eastern and the northern Brazilian shelves are respectively characterized by a southward transport driven by the Brazil Current (BC), and a north-westward transport driven by the North Brazil Current (NBC), both with a strong ocean mesoscale activity^22,24,25^. So far, their influence on the demographic connectivity has been little investigated. Rudorff *et al*., for example, used a simple advective/diffusive model to demonstrate that spiny lobster populations from Western Africa and the South Atlantic are not connected by larval transport driven by mean geostrophic flow^26^. Another study based on larval dispersion and the demographic connectivity of groupers (*Mycteroperca sp*.) living in MPAs along the eastern Brazil continental shelf found that the connectivity is uni-directional, directed from north to south, following the mean flow of the BC^21^. So far, the demographic connectivity of reef fishes living in MPAs under the influence of these flow regimes has not yet been assessed.

The present study aims to determine the demographic connectivity of MPAs located around the oceanic islands and the northern and eastern Brazilian continental shelves (10° N to 23°S) based on the dispersal abilities of reef fishes of the genus *Sparisoma*. The analysis is focused on the seasonal and interannual variability of mortality and recruitment rates under the influence of different current systems. We also discuss the likely impacts of larger recruitment areas (LRAs) on the demographic connectivity between MPAs. These LRAs are used here to assess the relative importance of non-protected areas as recruitment sites of larvae produced in MPAs.

## Results

**Figure 1:**
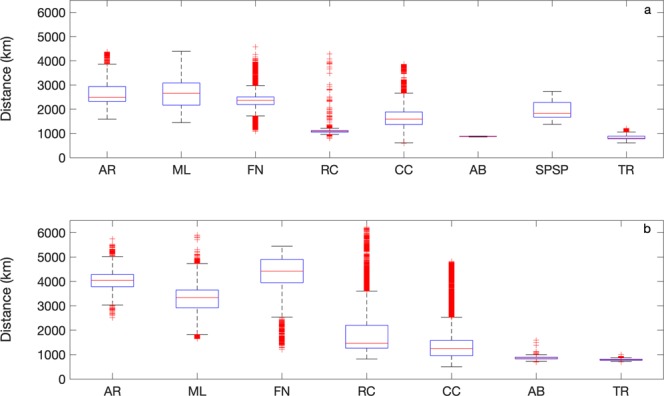
Boxplots of the average distances travelled by larvae for the summer (**a**) and winter (**b**) experiments for each spawning (MPA) site: Atol das Rocas (AR), Parcel do Manuel Luis (ML), Fernando de Noronha Archipelago (FN), Recife dos Corais (RC), Costa dos Corais (CC), Abrolhos (AB), São Pedro and São Paulo Archipelago (SPSP), and Trindade and Martim Vaz islands (TR). Red lines are median values and red crosses are outliers. Sites where all larvae died throughout the simulations were omitted.

The larvae that were spawned during the summer generally travelled a shorter total distance compared to those spawned during the winter (Fig. 1, Supplementary Figs S2 and S3). During the winter, larvae showed a longer linear drift with a tendency for Atol das Rocas (AR), Parcel do Manuel Luis (ML) and Fernando de Noronha (FN) larvae to travel longer distances due to the influence of the NBC and the North Equatorial Counter Current (NECC). This behaviour was more evident in the years 2008 to 2011 than from 2013 to 2015. Simulations show that, overall, total distances are less variable in summer than in winter (Fig. 1), with some larvae moving to the north and others moving to the south, influenced by the bifurcation of the southern branch of the South Equatorial Current (sSEC).

**Figure 2:**
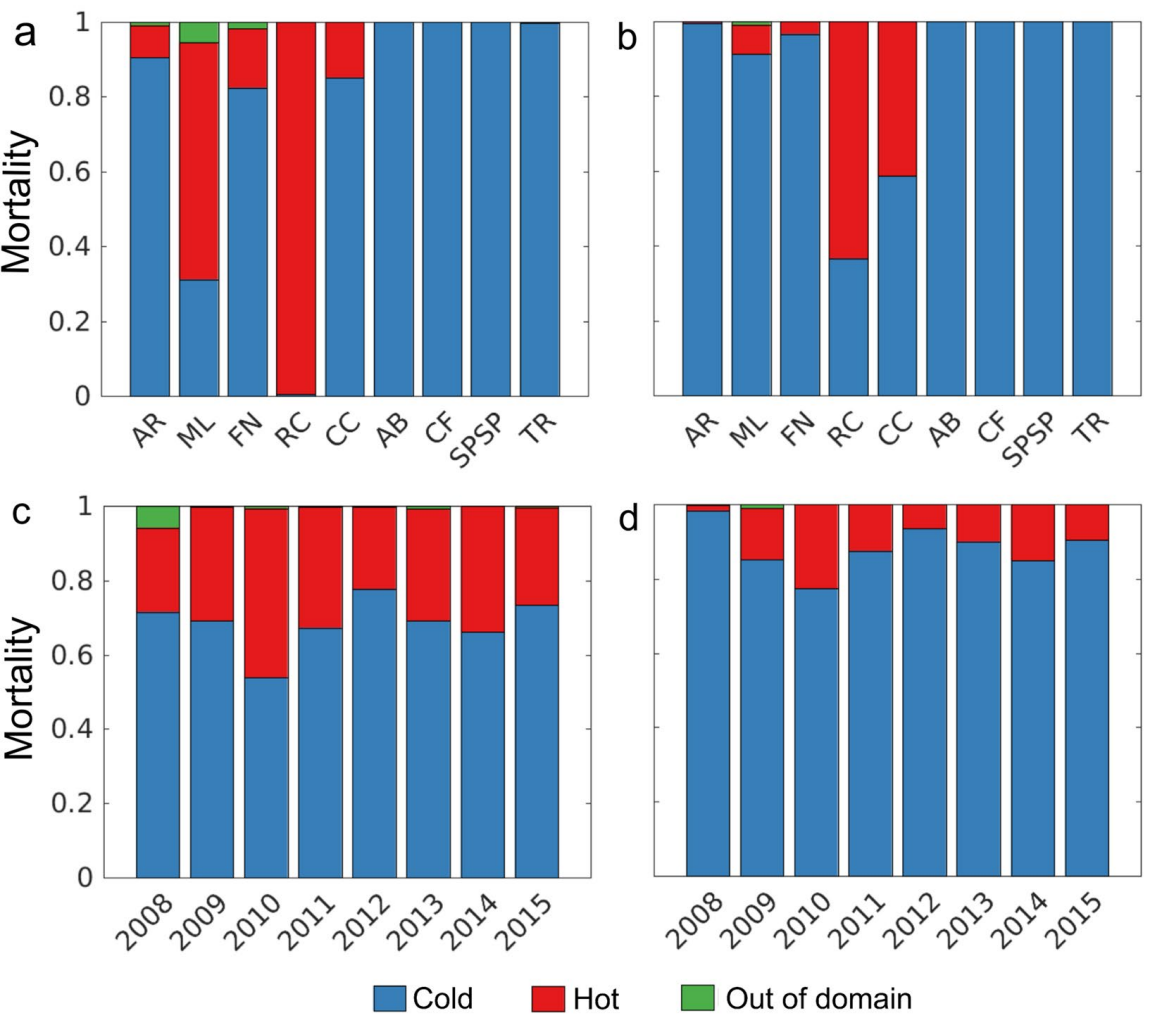
Total mortality partitioned in mortality by temperature and advection for summer (**a**,**c**) and winter (**b**,**d**). Mortality by temperatures higher than 30 °C are in red, by temperatures lower than 24 °C in are in blue and particles that were advected to outside the model domain are shown in green. Where: Atol das Rocas (AR), Parcel do Manuel Luis (ML), Fernando de Noronha Archipelago (FN), Recife dos Corais (RC), Costa dos Corais (CC), Abrolhos (AB), Arraial do Cabo and Cabo Frio (CF), São Pedro and São Paulo Archipelago (SPSP), and Trindade and Martim Vaz islands (TR).

Total mortality rates (Fig. S1) were significantly higher during the summer compared to winter (*p* = *0.0379*) and varied among the different spawning sites, but interannual variability was not significant. Summer and winter mortality were mainly caused by lethal low temperature (Fig. 2). Mortality by lethal high temperature was more frequent in the MPAs of Recife dos Corais (RC) and ML; during the summer and during the winter it was concentrated at RC and Costa dos Corais (CC). Mortality caused by advection of particles outside the model domain was one order of magnitude lower than mortality by temperature. Temperature-related mortality was higher in most of the northern MPAs, such as ML, AR and FN, being highly variable during the summer.

**Figure 3:**
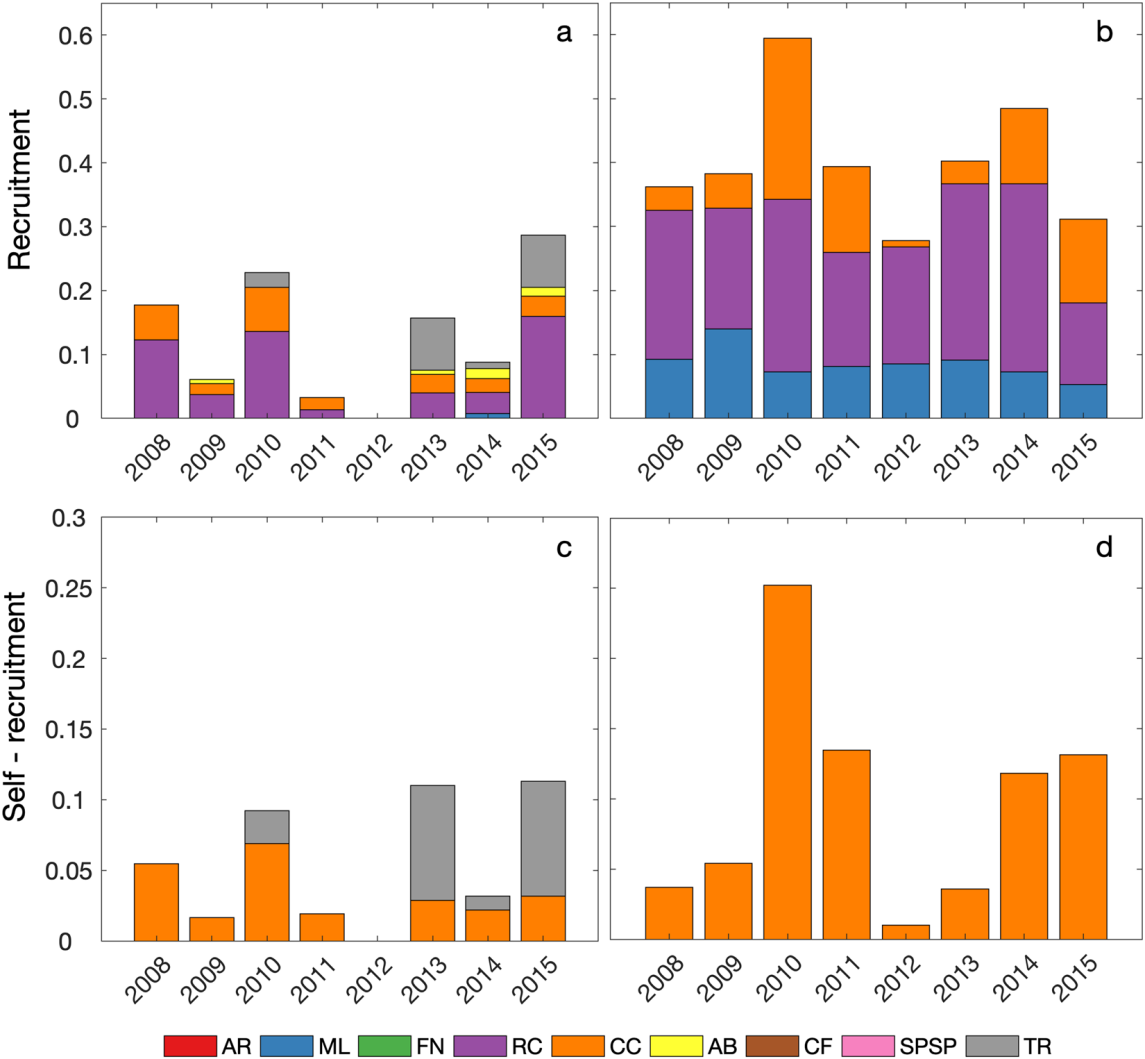
Recruitment and self-recruitment rates in each MPA for summer (**a,c**) and winter (**b,d**), respectively, grouped by year. Coded by colour: Atol das Rocas (AR), Parcel do Manuel Luis (ML), Fernando de Noronha Archipelago (FN), Recife dos Corais (RC), Costa dos Corais (CC), Abrolhos (AB), Arraial do Cabo and Cabo Frio (CF), São Pedro and São Paulo Archipelago (SPSP), and Trindade and Martim Vaz islands (TR).

**Figure 4:**
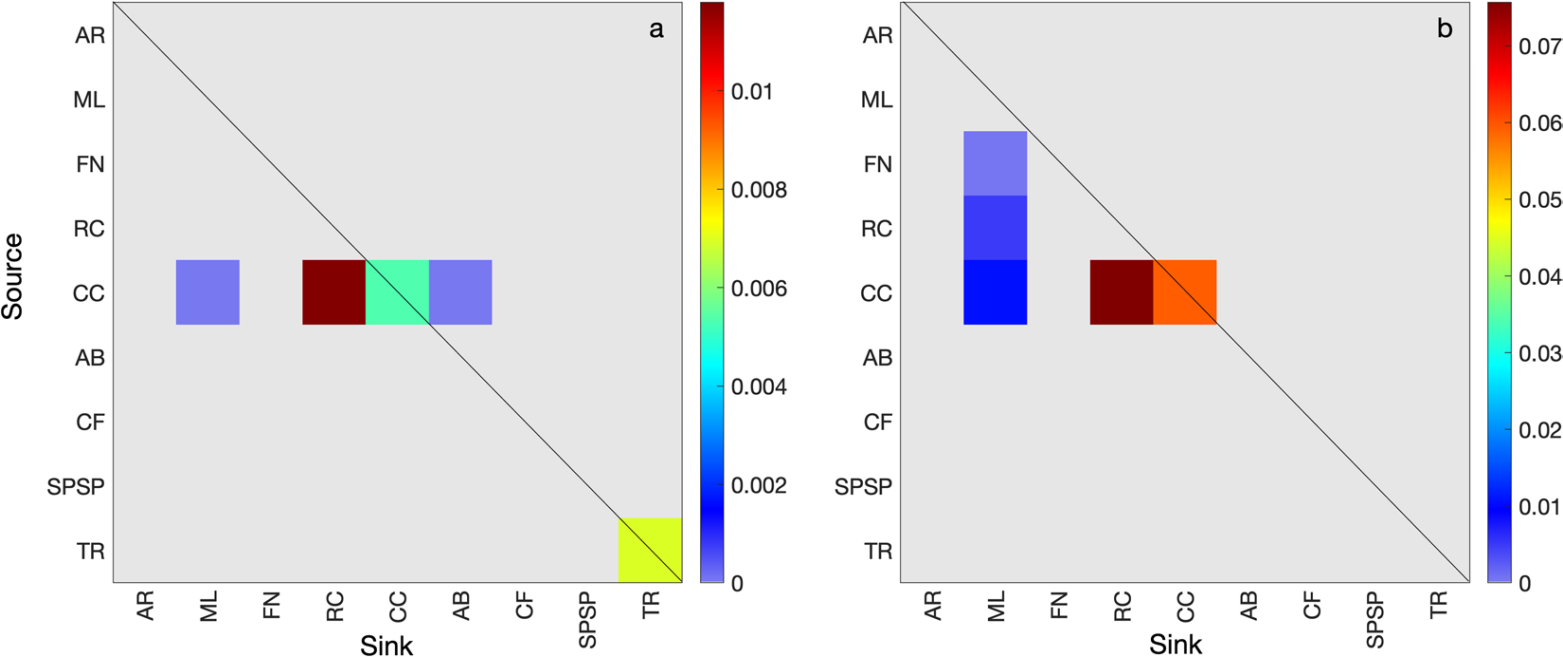
Transition probability matrix for the summer (**a**) and winter (**b**) considering the five coastal areas and the four oceanic islands. The five coastal MPAs: Parcel do Manuel Luis (ML), Recife dos Corais (RC), Costa dos Corais (CC), Abrolhos (AB) and Arraial do Cabo and Cabo Frio (CF); and the four oceanic islands: São Pedro and São Paulo Archipelago (SPSP), Atol das Rocas (AR), Fernando de Noronha Archipelago (FN) and Trindade and Martim Vaz islands (TR). Note the different colour scales for summer and winter.

The resulting recruitment and self-recruitment rates obtained from the eight-year simulation were low for summer and winter spawning (Fig. 3), which is not surprising given the relatively long distances travelled by the surviving larvae. Recruitment rates between 2008 and 2015 were close to zero in most sites except for RC, CC, ML and Trindade Archipelago (TR) (summer only) (Fig. 3a,b) and self-recruitment was non-zero for CC (summer and winter) and TR (summer only) (Fig. 3c,d). Most spawning sites presented no recruitment (or self-recruitment) and the interannual variability of recruitment rates in sites where it occurred can be up to one order of magnitude, as seen in RC and CC for winter spawning, or even a six-fold increase at RC for summer spawning. Important seasonal changes emerge from the high mortality/low recruitment scenario as shown in the seasonal transition probability matrices of Fig. 4. The spawning site CC was the only source of larvae for northern ML, RC and southern Abrolhos (AB) MPAs in the summer. During the winter, CC, RC and FN were source sites for northwesternmost site ML. Self-recruitment (diagonal line in Fig. 4) for summer spawning was only detected in CC and TR, while CC experienced self-recruitment also in the winter.

**Figure 5:**
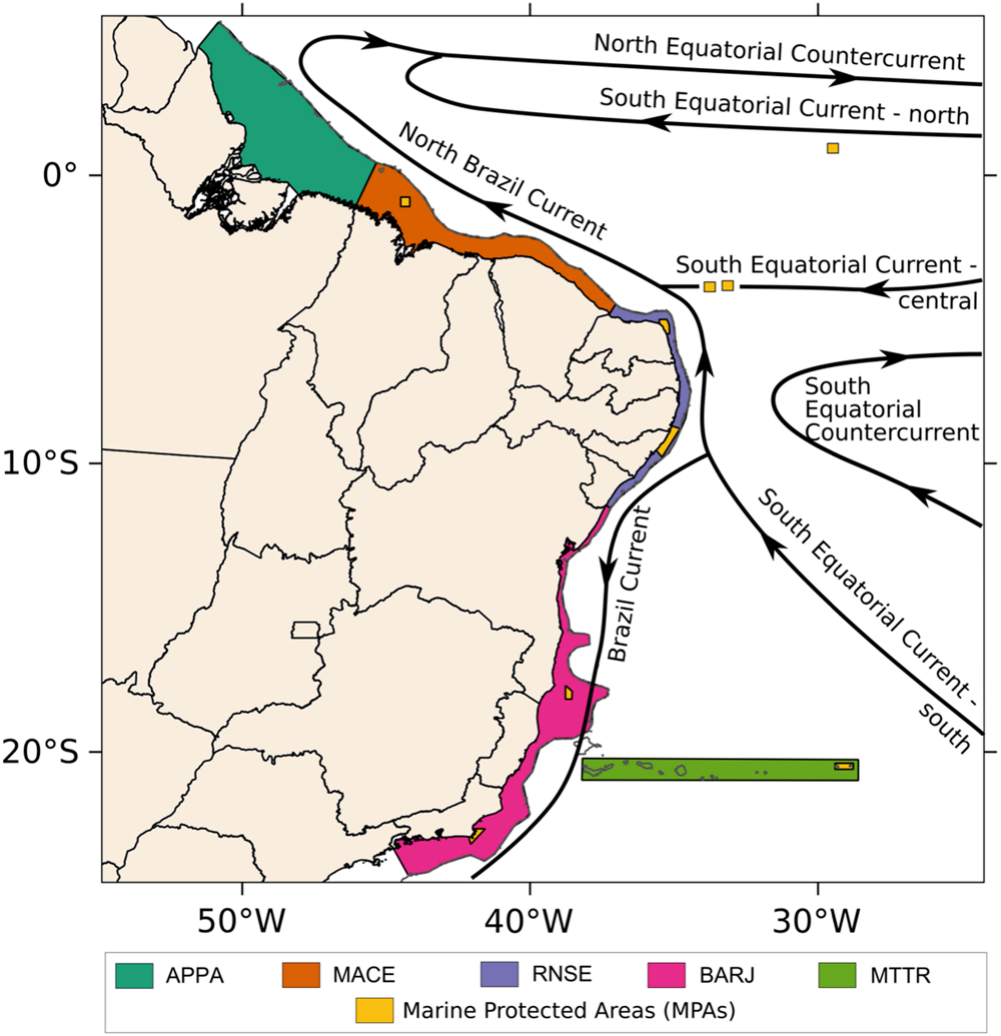
Map showing the Large Recruitment Areas (LRA) in the continental shelf and the main surface currents. Where APPA (dark green) is the region between the states of Amapá and Pará, MACE (orange) is the region between Maranhão and Ceará states, RNSE (violet) is the region between Rio Grande do Norte and Sergipe states, BARJ (pink) is the region between the states of Bahia and Rio de Janeiro and MTTR (light green) is the region of the Trindade and Martin Vaz islands and its seamounts. In yellow are the Marine Protected Areas.

**Figure 6:**
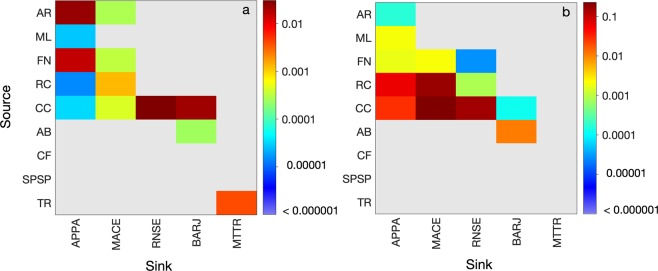
Transition probability matrix for the summer (**a**) and winter (**b**) considering as spawning areas the coastal areas and the oceanic islands and as recruitment sites the Large Recruitment Areas (LRAs). Where: AR – Atol das Rocas, ML – Parcel do Manuel Luis, FN – Fernando de Noronha Archipelago, RC – Recife dos Corais, CC – Costa dos Corais, AB – Abrolhos, CF – Arraial do Cabo and Cabo Frio, SPSP – São Pedro and São Paulo Archipelago, and TR – Trindade and Martim Vaz islands. The LRAs are APPA – Amapá to Pará region, MACE – Maranhão to Ceará region, RNSE – Rio Grande do Norte to Sergipe region, BARJ – Bahia to Rio de Janeiro region, and MTTR – Vitória-Trindade chain. Note the different colour scales for summer and winter.

Our results showed that particles reach other sites that are not currently protected by MPAs but could provide suitable recruitment conditions. So, we complemented our analysis by defining LRAs and we recalculated the connectivity (based on the same simulations) for five new areas (Fig. 5) to assess if these areas would be supported by the MPA’s and positively impact the demographic connectivity of *Sparisoma*. These new areas were defined based on the main surface flow domains depicted from our oceanic model results (Fig. 5), as follows: Amazon river outflow (APPA – continental shelf area from Amapá state to Pará state), NBC (MACE – continental shelf area from Maranhão state to Ceará state), bifurcation of the sSEC (RNSE – continental shelf area from Rio Grande do Norte state to Sergipe state), BC (BARJ – continental shelf area from Bahia state to Rio de Janeiro state), and Vitória-Trindade seamount chain (MTTR), including Trindade island. We observed higher connectivity rates compared to those between the original MPAs for the winter, whereas maximum connectivity figures in summer were very close to what was found for the original MPAs (Fig. 6). The number of potential sites for recruitment of eggs spawned in CC increased, specially to the north (MACE) and south (BARJ) of the domain (Fig. 6). However, the most relevant difference was the new source sites that emerged from the LRA approach, such as RC, FN, ML and AR for the summer spawning and ML, AR and AB for the winter spawning. This suggests that the new recruitment site MACE could possibly act as an important stepping stone between APPA and RNSE. An important southward expansion of potential recruitment sites was also identified within the southernmost BARJ region.

## Discussion

Demographic connectivity for long distance dispersal of *Sparisoma* in the tropical South Atlantic indicates that populations living in the present-day MPAs are highly isolated from each other (Fig. 1). The recent enlargement of two deep-ocean MPAs determined by the Brazilian government did not contribute to the development of an actual MPA network, because no new reef areas were involved and it is unlikely that it would contribute to the demographic connectivity^27^. Assuming a worst-case scenario where MPAs are the only source and sink areas available, the high mortality and low recruitment rates suggest that these populations are probably submitted to a high risk of local extinction and low probability of being rescued by other populations (Fig. 4) and are behaving like closed systems^19^. This assumption may not be realistic because there are other non-protected recruitment areas that could support viable populations (Fig. 6). However, in a what-if scenario where MPAs depend only on each other for population replenishment of *Sparisoma* recruits, our results clearly show that the present number and geographical location of MPAs, specially to the south, do not play the expected role in the conservation of this genus, as far as demographic connectivity is concerned.

Besides PLD, the behaviour of fish larvae can also influence their dispersion within short spatial scales and contribute to self-recruitment. In a model sensitivity experiment published elsewhere, the inclusion of horizontal and vertical movements of larvae at an early stage of development increased recruitment and connectivity^18,20^. So, by explicitly considering important biological characteristics such as spawning period, PLD, egg buoyancy, diel vertical migration, lethal water temperature and high (<10 km) horizontal resolution resolution we expect to have included the necessary level of realism in our simulations. However, we did not consider swimming and orientation capabilities of fish larvae in different periods of the PLD. These may vary among individuals of the same species and even within the same developmental stage^28^, making it difficult to incorporate these capabilities into biophysical models.

The strong seasonality of the upper ocean in the tropical Atlantic is characterized by a net northward heat transport between November-April, which probably contributed to the overall high mortality of eggs and larvae during the summer by high lethal temperatures. Mortality by high lethal temperature occurred in the spawning sites of ML and RC (Figs 2 and S1) and the mechanism that may have caused this increase in the surface temperature is the seasonal elevation of the thermocline^29^. However, in the oceanic islands of AR, FN, São Pedro and São Paulo archipelago (SPSP) and TR, the mortality tends to be higher during the winter (Fig. 2). Mortality by low water temperature in SPSP during the winter is likely to be influenced by the western tip of the equatorial upwelling^30^. All eggs spawned in Cabo Frio and Arraial do Cabo (CF) also died by lethal low temperature in the initial time steps of the simulations (summer and winter) due to the occurrence of Ekman-induced upwelling of the cooler South Atlantic Central Water (SACW, temperature <20 °C)^31^, which is more frequent during the summer. The negative model temperature bias (up to −2 °C) around the equator, compared with satellite data is, in fact, slightly larger in winter (Fig. S4) around SPSP. However, mortality by lethal low temperature is also prevalent even where model temperature bias is zero or slightly positive during winter (e.g., in CC, AB and ML) and summer (e.g., in AR and FN).

The seasonal and interannual surface circulation variability of the tropical Atlantic is an important driver of the regional connectivity by conditioning the recruitment pattern. The seasonal variability of the sSEC bifurcation position exerts an important role in the meridional (southward) transport of individuals. The mean latitudinal position of the sSEC bifurcation is highly correlated to the position of the Intertropical Convergence Zone (ITCZ) position and the wind stress curl, with a minimum (−4 × 10^−8^ Nm^−3^) in July and a maximum (2 × 10^−8^ Nm^−3^) in November^32^. During the austral spring/summer, the bifurcation is on its northernmost position (~10°S), associated with a positive wind stress curl, causing the bifurcation to be positioned at lower latitudes. In this scenario, the southward water transport of BC increases due to anomalous anticyclonic circulation^32^, allowing the recruitment in AB of particles originating from CC located to the north (Fig. 4). During the winter, AB does not receive any particle at all, which coincides with the southernmost position of the sSEC bifurcation and a decrease of the BC southward transport in the region. The importance of CC to the maintenance of populations to the north (RC) and to the south (AB) is strongly linked to the seasonal march of the ITCZ, which also modulates the climate in the northeast of Brazil^33^ and plays a strategic role in the conservation framework of the MPAs in Brazil. The contribution of RC to the winter recruitment in ML suggests that both CC and RC could be considered as targets for a largescale protected area complex, responsible for promoting a north-south connectivity either during summer or winter.

Self-recruitment was extremely rare, occurring in TR (0.69%) and CC (0.54%) during the summer and only in CC (5.93%) during the winter. Self-recruitment in these cases occurred as a result of strong and persistent ocean mesoscale activity that favours larval retention. Self-recruitment in reef fish populations may vary from zero to 65%^34^. It is also argued that short distance transport and self-recruitment are more important than long distance dispersal for the connectivity in the ecological time scale (from hours to years). Our simulations have shown to be able to capture both processes and the lack of widespread self-recruitment further highlights the fragility of *Sparisoma* populations living in the studied MPAs. Fine-scale reef topography, active selection of spawning sites by adult fishes and behavioural traits can all enhance self-recruitment but have not been explicitly incorporated in our study. Therefore, our results should be considered as a conservative scenario and actual self-recruitment may be higher than shown in our simulations.

Based on the low connectivity scenario described above, we tested the concept of LRAs (Fig. 5) based on recommendations for a global conservation agenda as the Aichi 11^34–36^. The results reinforce the importance of RC and CC, located within the new large unit called RNSE, as an important source of larvae connecting northern and southern sites (Fig. 6). The emergence of the oceanic sites of FN and AR as sources of larvae during both summer and winter spawning also highlights their relative importance for the northern and central (APPA, MACE and RNCE) coastal populations. Also, AB becomes a relevant local player as it is, together with CC, the only source of larvae for the southernmost region BARJ. Information on species movement patterns can help conservation practitioners to define focal species for protection and a reserve configuration which is capable of protecting them^34^. Considering that biological requirements of *Sparisoma*, used as a model species here, are common to other reef fish species, our results raise relevant concerns about the efficacy of present-day MPAs in conserving this group.

Thus, assuming that connectivity is low and self-recruitment is spatially restricted, how could *Sparisoma* populations be found in all the studied MPAs? We hypothesize that the crossing of oceanographic barriers facilitated by the NBC and NECC has allowed the migration of *Sparisoma* from Brazil to the Caribbean Province and West Africa, respectively^37^. Despite the widespread occurrence of the Scaridae family in the tropical South Atlantic, the only species of this family that occur in the oceanic islands are from the genus *Sparisoma*^38^. This supports the model results we presented here of the isolated condition of the studied islands driven by the surface flow regime.

The biogeography of reef fishes in the South Atlantic has long been considered a puzzle due to the complex distribution pattern of fish species compositions seen today^23^. The observed presence of vagrants suggests that in the long run species could reach distant reefs with the persistent use of stopovers along short routes. Reef fish species composition from the north-western and south-western Atlantic are similar, but they differ from the mid-Atlantic (Ascencion and St. Helena) and eastern Atlantic^1^.

It is possible that the extremely low connectivity observed in our simulations has already been in operation on the evolutionary (genetic) timescale and contributed to the high endemism of reef fish observed today. Annual mean wind stress, fresh water and heat exchange in the tropical Atlantic for the Oligocene (33.9 to 23.03 Ma) and Miocene (23.03 to 5.3 Ma), simulated by a fully coupled climate model, displayed patterns similar to the present-day conditions^39^. These are important drivers of the surface flow and SST variability in the tropical Atlantic that influence larval advection and survival.

In the case of *Sparisoma*, the relevant cladogenic event time is positioned between 8 to 2 Ma^1^, which includes the closure of the Isthmus of Panama. It is beyond the scope of the present study to discuss the biogeography of reef fishes, but the point raised above might be relevant to place the connectivity of MPAs in perspective. Especially in the time scales from years to decades, both strongly restricted connectivity among suitable habitats and ecomorphological adaptations^40^ may have contributed to the endemism.

Hence, empirical evidence and model results provide clear indications that the demographic connectivity of *Sparisoma* populations living in the MPAs of the tropical South Atlantic is extremely low and that this condition, together with long dispersion distances that separate MPAs, enhances their isolation. The high mortality rates and low recruitment point to a high risk of local extinction and low probability of being rescued by populations from other MPAs. It appears that the present MPAs by themselves are not capable of conserving populations of this genus and, by analogy, several other reef fish species. Although LRAs were not used as spawning sites in our simulation, it is clear that they behave as important sink areas. Based on that, we contend that protecting LRAs (similarly to Large Marine Protected Areas^20^) may improve connectivity not only between offshore islands and the coast, but also along the northern and southern limits of the tropical Brazilian coast. Our findings highlight a long-standing concern about the need to manage ecosystems outside MPAs as a necessary step to enhance their effectiveness in preserving important species^41^. The realistic assessment of the dispersal abilities of target species is important for a proper determination of spatial priorities based on demographic connectivity. It should be highlighted, however, that in a multi-species approach other important issues need to be considered for the selection of new areas for protection, such as habitat quality and the interplay of different dispersal (and connectivity) abilities^42^.

## Data and Methods

### Study area

**Figure 7:**
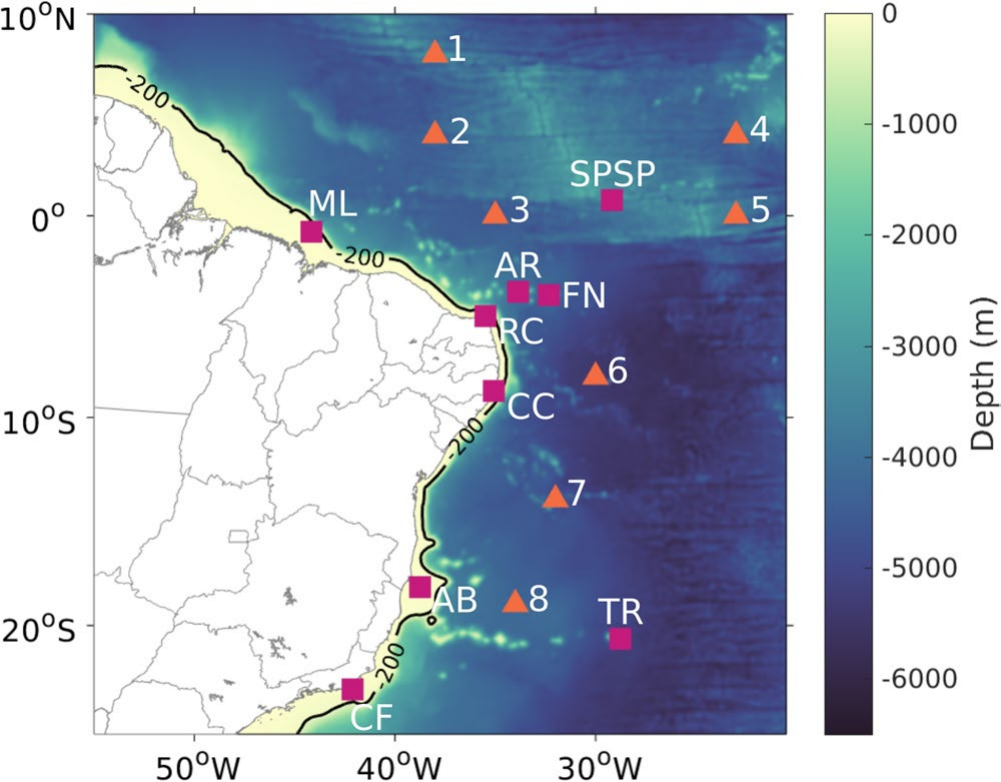
Study area with the marine protected areas location, shown by the pink squares, used for spawning and recruitment of Sparisoma. Five areas are in the continental shelf: ML – Parcel do Manuel Luis, RC – Recife dos Corais, CC – Costa dos Corais, AB – Abrolhos and CF – Arraial do Cabo and Cabo Frio; and four are oceanic islands: SPSP – São Pedro and São Paulo Archipelago, AR – Atol das Rocas, FN – Fernando de Noronha Archipelago and TR – Trindade and Martim Vaz islands. Prediction and Research Moored Array in the Tropical Atlantic (PIRATA) buoys used for the ROMS model evaluation, shown by orange triangles, where: 1–8°N 38°W, 2–4°N 38°W, 3–0° 35°W, 4–4°N 23°W, 5–0° 23°W, 6–8°S 30°W, 7–14°S 32°W and 8–19°S 34°W.

The study region (Fig. 7) is in the Brazilian coast and contains five regions on the continental shelf: Manuel Luiz (ML), Recife de Corais (RC), Costa dos Corais (CC), Abrolhos (AB) and Arraial do Cabo and Cabo Frio region (CF); and four oceanic islands: Atol das Rocas (AR), Fernando de Noronha Archipelago (FN), São Pedro and São Paulo Archipelago (SPSP) and Martim Vaz and Trindade Archipelago (TR). These sites correspond to all federal MPAs in the tropical Brazilian coast with reefs and they were used as recruitment and spawning sites (Table S6).

### Models and auxiliary data

Physical processes were modelled using the Regional Ocean Modeling System (ROMS)^43^ with a numerical model domain extending from 55°S to 10°N and from 70°W to 20°W. A horizontal resolution of 1/12° was used (approximately 9,2 km) with 30 sigma levels in the vertical. The model was forced using 6-hourly atmospheric fields derived from the Climate Forecast System Reanalysis (CFSR^44^) at the surface, and 5-day ocean fields of temperature (T), salinity (S), zonal current speed (u), meridional current speed (v) and sea surface height (SSH) obtained from the Simple Ocean Data Assimilation reanalysis (SODA^45^) as lateral oceanic boundary conditions. ROMS was *spun up* for 4 years starting in 2000. After that, monthly outputs from 2004–2015 were stored to evaluate the model performance. At the surface, ROMS model outputs were evaluated and compared to objective analyses derived from the Operational Sea Surface Temperature and Sea Ice Analysis (OSTIA)^46^ and the Ocean Surface Current Analyses Real-time (OSCAR)^47^ (Supplementary Fig. S4). The model temperature and salinity at subsurface depths were validated against *in situ* profiles from the Prediction and Research Moored Array in the Tropical Atlantic moorings (PIRATA)^48^ (Supplementary Fig. S5).

Specifically, for the years 2008 to 2015, hourly outputs from January to February (summer) and July to August (winter) were stored, including the main physical variables (e.g. T, S, u, v) to be used as an input for the lagrangian dispersion experiments. Similar model configurations have been used to the south-west Atlantic ocean for physical^49,50^ and biophysical studies^21,51^.

The lagrangian dispersion experiments were performed with the individual based model (IBM) Ichthyop v.3.3^52^ using the reproductive characteristics of the grazer parrotfish genus *Sparisoma* (Scaridae) obtained from the literature and detailed in Table 1. Spawning can happen at any time during the year but tends to concentrate during summer and winter and the eggs are released in the first meters of the water column^53^. The hydrodynamic and the IBM models were coupled offline in which Ichthyop used the physical variables from the ROMS hourly outputs to track larvae dispersal and survival throughout the model domain.

**Table 1:** Table with *Sparisoma* biological characteristics used in Ichthyop v.3.3. to perform the experiments for the summer and winter for the period from 2008 until 2015.

Variable	Value
Total number of released particles	70 000
Pelagic larval Duration	58 days^3,5^
Coastline behaviour	Bouncing
Advection method	Runge Kutta 4
Turbulent dissipation rate	1 × 10^−9^ m^2^/s^3 ^^54^
Egg density	0.0089 g/cm^3^^55^
Egg hatching age	24 hours^56^
Diel vertical migration depth	58 (day) to 27 (night) m^57^
Sunset and sunrise	6:00–18:00
Temperature optimum range	24–30 °C^58–60^

The model scenarios included sixteen experiments performed for each of the eight summers and winters between 2008 and 2015, with one spawning event of 70.000 eggs for each experiment. Eggs were proportionally distributed with larger MPAs releasing more eggs than smaller MPAs (Table S6), allowing results to be comparable. MPAs were considered as spawning and recruitment sites (Fig. 7) to analyse if their role to preserve the species is being accomplished. We used a conservative PLD of 58 days, which is well within the range of values for this species^3^, in all experiments. This experiment set up was chosen to produce the most realistic dispersal and connectivity scenarios, while keeping computational cost at a manageable level.

### Mortality, recruitment and connectivity

The Ichthyop model outputs were used to calculate the distance travelled by larvae, mortality, recruitment and self-recruitment. Mortality was calculated using Eq. 1 and it computes every egg or larva that either died by advection off the optimum temperature range (Table 1, temperature optimum range) or was advected outside the domain. Self-recruitment is defined as larvae that are recruited in the same site where they were spawned and was calculated using Eq. 2. Recruitment was considered when larvae were found inside the MPAs by the end of simulations, as shown in Eq. 3. Note that self-recruitment is treated here as a special case of recruitment as both are computed in Eq. 3. Once a larva reached an MPA (spawning sites shown in Fig. 7 are also recruitment sites) at any time step during the simulation, it stopped moving and it was then considered as a recruit to that MPA.

1 $$Mortalit{y}_{t}=\frac{{\sum }_{t}d}{N}$$

2 $$Self \mbox{-} recruitmen{t}_{t,i}=\frac{{\sum }_{t}\,{c}_{ii}}{{\sum }_{t}{N}_{i}}$$

3 $$Recruitmen{t}_{t,i}=\frac{{\sum }_{t}{c}_{ij}+{\sum }_{t}{c}_{ii}}{{\sum }_{t}{N}_{s}}$$

where:

*d* – particles that died by advection outside the temperature optimum range or to outside the domain;

*N* – total number of eggs spawned in the domain;

*t* – integration time representing each summer and winter of each year;

*c*_*ii*_ – particles that were spawned in the MPA *i* and were recruited in the same MPA;

*c*_*ij*_ – particles that were spawned in the MPA *j* and were recruited in the MPA *i*.

*N*_*i*_ – number of eggs spawned in MPA *i*;

*N*_*s*_ – number of eggs spawned in the sources of particles to MPA *i*;

The connectivity matrix, or the transition probability matrix, describes the probability of a surviving individual to move from the spawning site to the recruitment site. This is calculated using the number of larvae originated from MPA “*j*” which was recruited to MPA “*i*”, divided by total of individuals spawned in MPA “*j*”.

## Supplementary information

Suplementary Information

## Data Availability

The datasets generated during and/or analysed during the current study are available from the corresponding author on reasonable request.
