# Supplementary material for: Low connectivity compromises the conservation of reef fishes by marine protected areas in the tropical South Atlantic
Source: Sci Rep. 2019 Jun 14;9:8634. doi: 10.1038/s41598-019-45042-0 (PMC6572763; doi:10.1038/s41598-019-45042-0)
Supplement: Supplementary file 1 — Suplementary Information [file 41598_2019_45042_MOESM1_ESM.pdf]

Low connectivity compromises the conservation of reef fishes by marine protected areas in the tropical South Atlantic

Clarissa Akemi Kajiya Endo[1+*], Douglas Francisco Marcolino Gherardi[1+], Luciano Ponzi Pezzi[1+], Leonardo Nascimento Lima[2+]

[1] National Institute for Space Research (INPE), Remote Sensing Department, São José dos Campos, 12227-010, Brazil

[2] Euro-Mediterranean Center on Climate Change (CMCC), Ocean modeling and Data Assimilation Division, Bologna, 40127, Italy

*clari.endo@gmail.com

[+] these authors contributed equally to this work

Supplementary information:

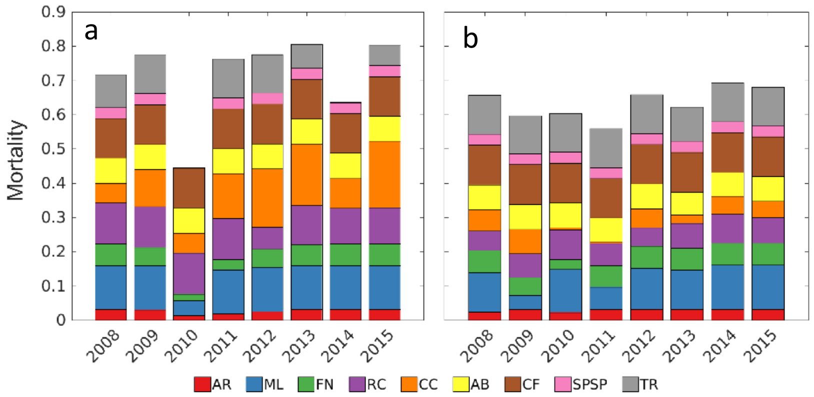

Figure S1. Total mortality for the summer (a) and winter (b) of each year and each colour represent a spawning site.

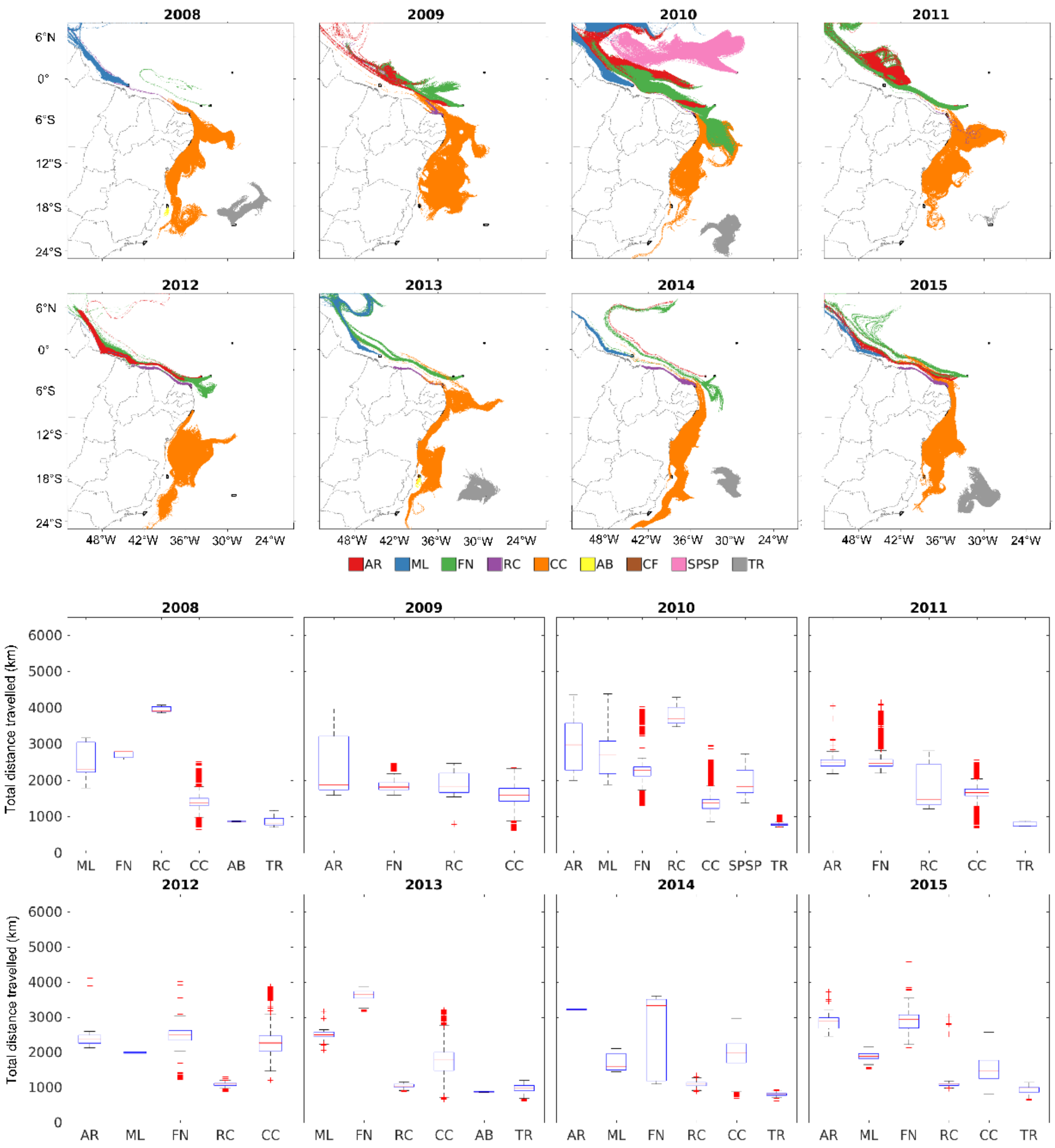

Figure S2. Maps showing the total drift and total distances travelled by larvae for the summer experiments for each year. Central mark indicates the median, the whiskers indicate extreme data points and red crosses are outliers. The boxplots allow the interannual comparison of total drifts for each spawning (MPA) site. Those sites where all larvae died in the initial time steps of simulations were omitted.

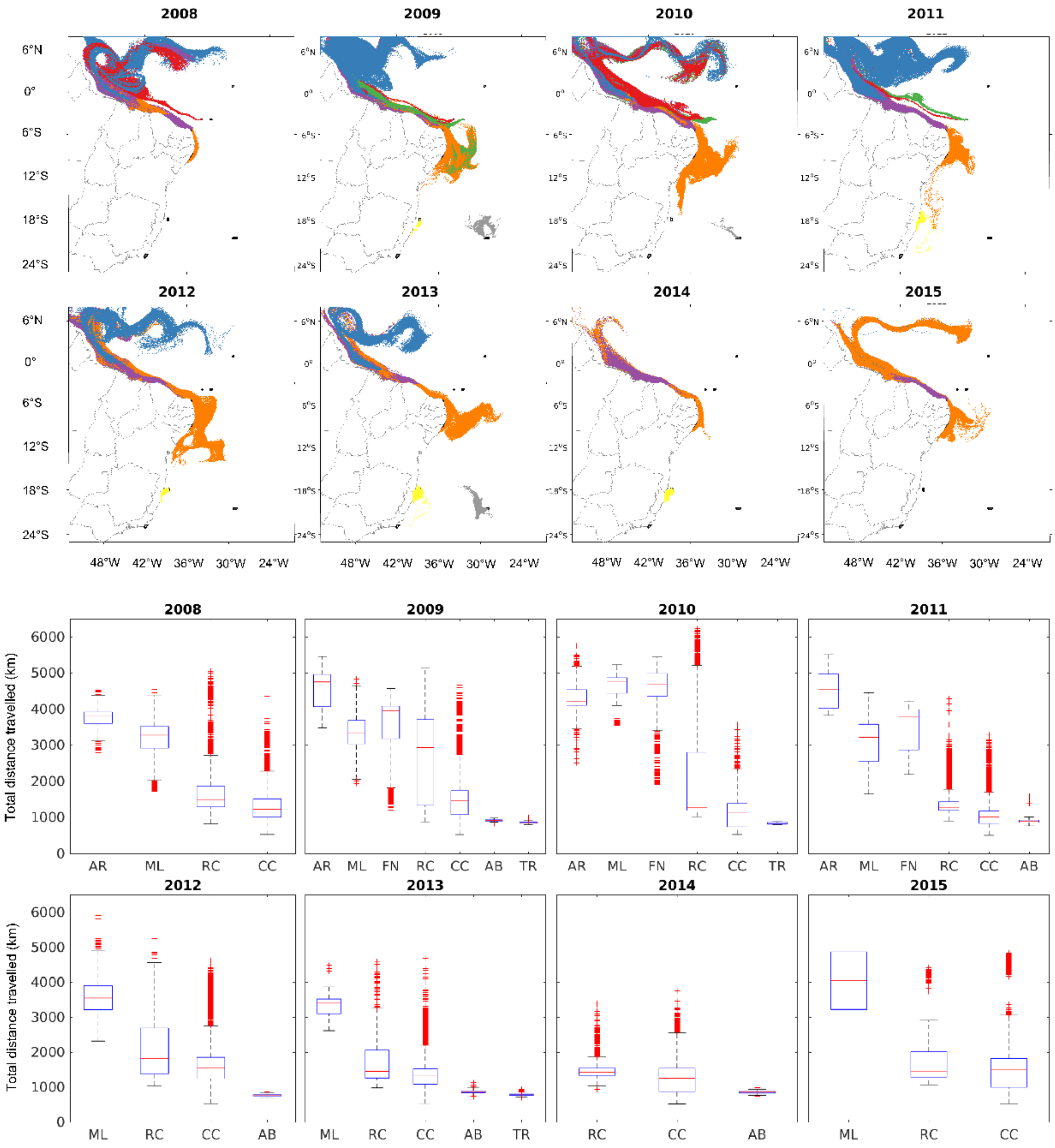

Figure S3. The same as S2 for the winter experiments.

Sea surface temperature fields for the summer and winter from ROMS and from OSTIA analysis.

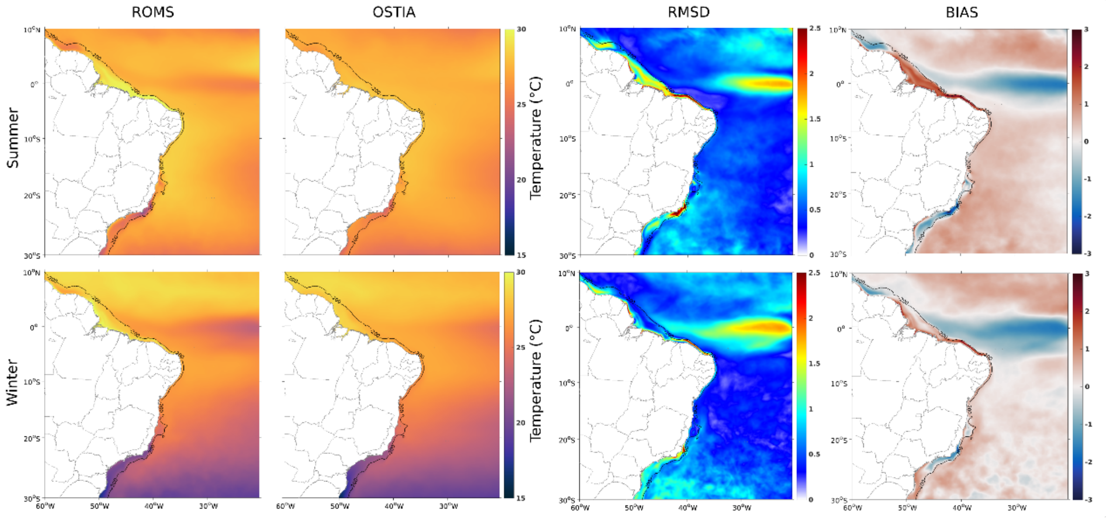

Sea surface currents meridional and zonal components from ROMS outputs and from OSCAR.

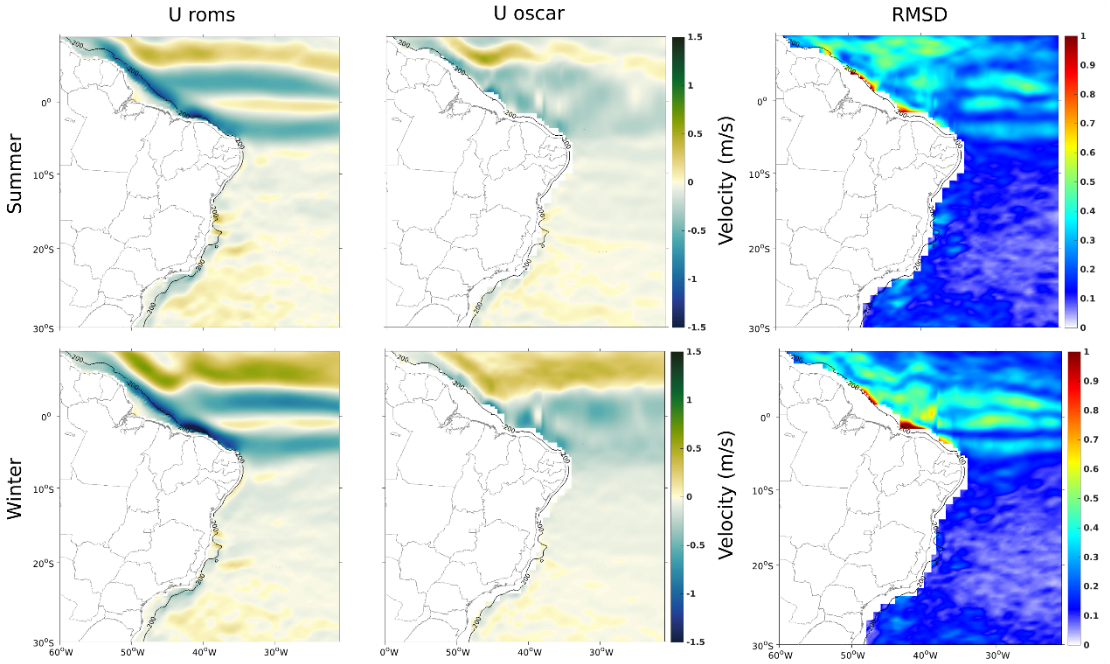

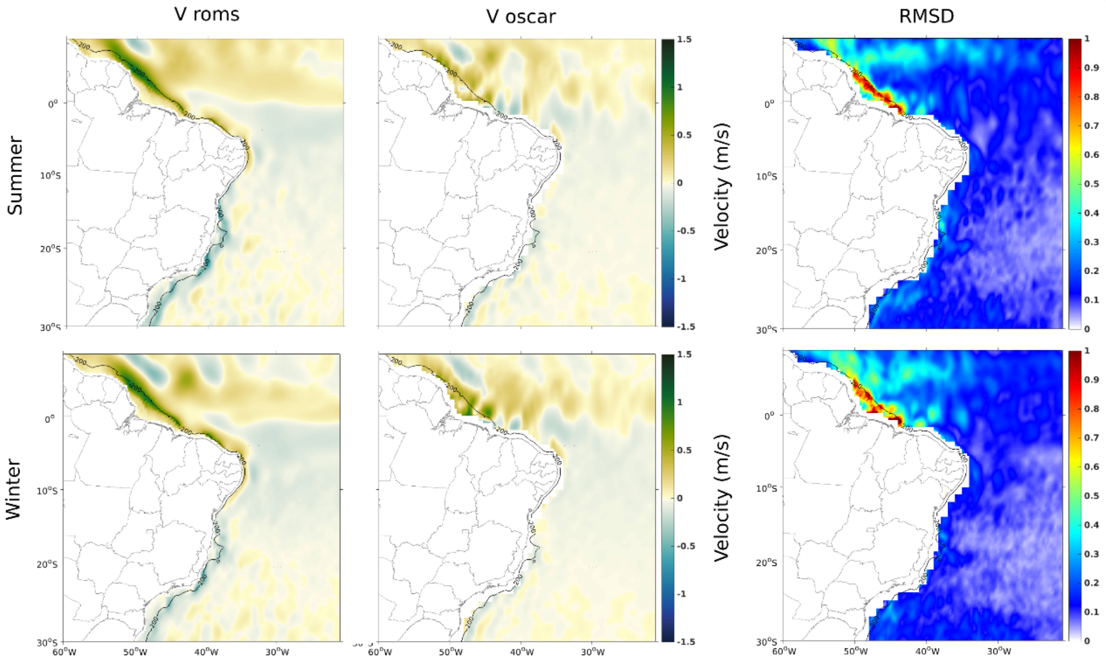

Figure S4. Comparison of remote sensing data with hydrodynamic model results based on root mean square deviation and model bias.

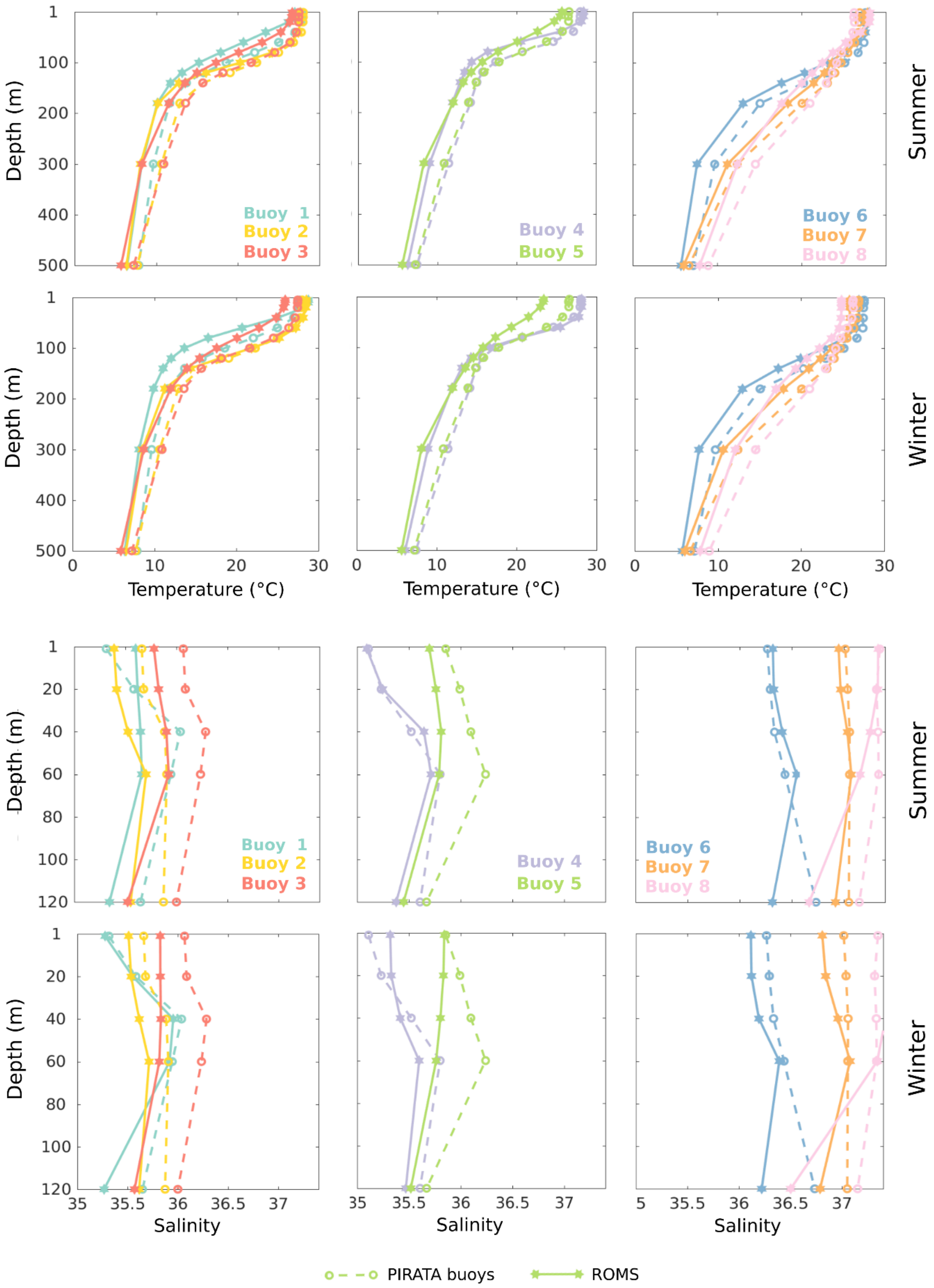

Figure S5. Comparison of temperature and salinity vertical profiles from ROMS outputs and PIRATA buoys in the model domain. The stars with the continuous line correspond to the values calculated by ROMS and the hollow circles with the dotted lines correspond to the PIRATA buoys measurements.

Marine protected area	Area (km^2)
**Atol das Rocas (AR)**	308.67
**Parcel do Manuel Luis (ML)**	257.59
**Fernando de Noronha Archipelago (FN)**	447.19
**Recife dos Corais (RC)**	1507.29
**Costa dos Corais (CC)**	3995.39
**Abrolhos (AB)**	992.42
**Cabo Frio and Arraial do Cabo (CF)**	1616.65
**Sao Pedro and Sao Paulo Archipelago (SPSP)**	125.65
**Trindade and Martim Vaz islands (TR)**	2299.90

Table S6. Table with the marine protected areas in the Brazilian coast used in this study and their respective area in the biological model experiments.
